# A novel kidney disease index reflecting both the albumin-to-creatinine ratio and estimated glomerular filtration rate, predicted cardiovascular and kidney outcomes in type 2 diabetes

**DOI:** 10.1186/s12933-022-01594-6

**Published:** 2022-08-22

**Authors:** Hertzel C. Gerstein, Chinthanie Ramasundarahettige, Alvero Avezum, Jan Basile, Ignacio Conget, William C. Cushman, Gilles R. Dagenais, Edward Franek, Mark Lakshmanan, Fernando Lanas, Lawrence A. Leiter, Nana Pogosova, Jeffrey Probstfield, Peter J. Raubenheimer, Matthew Riddle, Jonathan Shaw, Wayne H.-H. Sheu, Theodora Temelkova-Kurktschiev, Ibrahim Turfanda, Denis Xavier

**Affiliations:** 1 Population Health Research Institute, McMaster University and Hamilton Health Sciences, Hamilton, Canada; 2 International Research Center, Hospital Alemao Oswaldo Cruz, Sao Paulo, Brazil; 3 Medical University of South Carolina, Charleston, SC USA; 4 Endocrinology and Nutrition Department, University of Barcelona, Barcelona, Spain; 5 Department of Preventive Medicine, University of Tennessee Health Science Center, Memphis, TN USA; 6 Institut Universitaire de Cardiologie et de Pneumologie de Québec, Université Laval, Quebec, Canada; 7 Mossakowski Clinical Research Center, Polish Academy of Sciences, Warsaw, Poland; 8 Eli Lilly and Company, Indianapolis, IN USA; 9 Universidad de La Frontera, Temuca, Chile; 10 St. Michael’s Hospital, Li Ka Shing Knowledge Institute, University of Toronto, Toronto, Canada; 11 National Medical Research Center of Cardiology, Moscow, Russia; 12 University of Washington, Seattle, USA; 13 Department of Medicine, University of Cape Town, Cape Town, South Africa; 14 Department of Medicine, Oregon Health & Science University Portland, Oregon, USA; 15 Baker Heart and Diabetes Institute, Melbourne, Australia; 16 Division of Endocrinology and Metabolism, Department of Internal Medicine, Taipei Veterans General Hospital, Taipei, Taiwan; 17 School of Medicine, National Yang Ming Chiao Tung University, Taipei, Taiwan; 18 School of Medicine, National Defense Medical Center, Taipei, Taiwan; 19 Robert Koch Medical Center, Sofia, Bulgaria; 20 Eli Lilly and Company, Lilly Corporate Center, Indianapolis, IN USA; 21 St. John’s Research Institute, St. John’s National Academy of Health Sciences, Bangalore, India; 22 Department of Medicine, McMaster University, HSC 3V38, 1280 Main Street West, Hamilton, ON L8S 4K1 Canada

**Keywords:** Risk Factor, Kidney, Albuminuria, Cardiovascular Outcomes, Kidney Outcomes

## Abstract

**Background:**

The estimated glomerular filtration rate (eGFR) and the albumin-to-creatinine ratio (ACR) are risk factors for diabetes-related outcomes. A composite that captures information from both may provide a simpler way of assessing risk.

**Methods:**

9115 of 9901 Researching Cardiovascular Events with a Weekly Incretin in Diabetes (REWIND) participants with both an ACR and eGFR at baseline were included in this post hoc epidemiologic analysis. The hazard of higher baseline levels of 1/eGFR and natural log transformed ACR (calculated as ln [ACR × 100] to eliminate negative values) and their interaction for incident major adverse cardiovascular events (MACE), kidney outcomes, and deaths was estimated. The hazard of the geometric mean of these two baseline measures (the kidney disease index or KDI) was also assessed.

**Results:**

A non-linear relationship was observed between 1/eGFR and all three outcomes, and between ln [ACR × 100] and the kidney outcome. There was also a negative interaction between these two risk factors with respect to MACE and death. Conversely, a linear relationship was noted between the KDI and all three outcomes. People in the highest KDI fifth experienced the highest incidence of MACE, death, and the kidney outcome (4.43, 4.56, and 5.55/100 person-years respectively). C statistics for the KDI were similar to those for eGFR and albuminuria.

**Conclusions:**

The KDI combines the baseline eGFR and ACR into a novel composite risk factor that has a simple linear relationship with incident serious outcomes in people with diabetes and additional CV risk factors.

***Trial Registration*** clinicaltrials.gov NCT01394952.

**Supplementary Information:**

The online version contains supplementary material available at 10.1186/s12933-022-01594-6.

## Background

The estimated glomerular filtration rate (eGFR) and the degree of albuminuria measured by the albumin-to-creatinine ratio (ACR) are routinely measured in people with diabetes and are recognised as prognostic indices for kidney outcomes, cardiovascular outcomes, and death. Indeed, large meta-analyses and current guidelines support the combined use of both measures to identify people at highest risk of these outcomes [1, 2]. These guidelines are supported by later large prospective studies [3] that assess these risk factors as either continuous variables or as categorical variables focused on the stage of kidney disease (defined using various eGFR thresholds) and the presence of either microalbuminuria (defined as an ACR of 30–300 mg/g) or macroalbuminuria (defined as an ACR > 300 mg/g) [2]. These reports generally assessed these 2 risk factors independently and usually assumed a linear relationship between them and incident outcomes.

The Researching Cardiovascular Events with a Weekly Incretin in Diabetes (REWIND) trial recruited 9901 individuals of mean age 66 years (46% women) who had type 2 diabetes and additional cardiovascular risk factors, and randomly assigned them to the glucagon-like peptide 1 receptor agonist dulaglutide or placebo. Participants were followed for a median of 5.4 years during which the hazard of major adverse cardiovascular events (MACE) was significantly reduced by 12% with dulaglutide *versus* placebo. The availability of baseline albuminuria and an eGFR in 9115 REWIND participants, the long-term follow-up and the high number of kidney outcomes, cardiovascular outcomes and deaths provide an opportunity to assess the nature of the relationship of these 2 baseline measures to the incidence of these outcomes. It also presents an opportunity to determine whether combining these two measures into one index has a similar or stronger relationship to these outcomes than the components.

## Methods

As previously published [4, 5], the REWIND trial recruited people aged 50 or older with either newly diagnosed or established type 2 diabetes whose body mass index was ≥ 23 kg/m^2^, and whose HbA1c was 9.5% or less (with no lower limit) on stable doses of up to 2 oral glucose-lowering drugs with or without basal insulin between August 2011 and 2013. Exclusion criteria included an estimated glomerular filtration rate (eGFR) < 15 mL/min/1.73 m^2^. Participants were randomly assigned to weekly subcutaneous injections of either dulaglutide 1.5 mg or identical placebo and followed for a median period of 5.4 years for the occurrence of clinical outcomes. Annual laboratory assessments (measured at local laboratories) included serum creatinine and a urine albumin-to-creatinine ratio (ACR). The protocol was reviewed by Research Ethics Boards for 371 sites in 24 countries and all participants provided signed written informed consent.

### Outcomes

The primary outcome of the REWIND trial was the first occurrence of MACE comprising blindly adjudicated nonfatal myocardial infarction, nonfatal stroke, or death from cardiovascular or unknown causes. This post-hoc epidemiologic analysis focused on this outcome, total mortality, and a kidney composite outcome comprising new macroalbuminuria (i.e., a ACR > 33.9 mg/mmol), a sustained decline in eGFR of ≥ 40%, or chronic kidney replacement therapy.

### Model validation

Validation was done using data from 12,187 Outcomes Reduction with and Initial Glargine Intervention (ORIGIN) trial participants (mean age 64 years, 35% women) who had a baseline eGFR and ACR measurement and who were followed for a median of 6.2 years [6]. MACE and death from cardiovascular causes were assessed in ORIGIN using the same definition as the REWIND trial. The kidney composite outcome in that trial (based on measurements at baseline, 2-years and study end) was defined as the first occurrence of a doubling of serum creatinine, worsening of albuminuria category (from normal to microalbuminuria or clinical proteinuria, or from microalbuminuria to clinical proteinuria), chronic kidney replacement therapy or death due to kidney failure [7].

### Statistical analyses

This report describes epidemiologic analyses focused on the prognostic relevance of the eGFR and ACR, regardless of random assignment, and is restricted to participants who had both measurements recorded at baseline. Continuous variables were summarized using either the arithmetic mean and standard deviation or the median for which either interquartile or inter-decile (i.e., between the 10th and 90th percentile) ranges were reported. Categorical variables were summarized as counts with percentages and compared across categories using the chi-square statistic.

The eGFR was calculated at each visit using the Modification of Diet in Renal Disease equation [8] and was transformed prior to analyses by calculating the inverse of its value (i.e., 1/eGFR) so that higher levels would reflect worse kidney function. The skewed distribution of ACR was normalized by calculating the natural logarithm (ln) of each measurement. ACR values below the lower limit of detection of 0.1 mg/g were assigned this lower limit. Because the ACR could be less than 1 (for which the ln would be negative) the ACR values were multiplied by 100 prior to this transformation. These two indices of kidney status were analyzed separately and combined into a novel kidney disease index (KDI) by calculating the geometric mean of these two values for each participant [9]. Baseline characteristics and the incidence of the reported outcomes were presented overall and after dividing participants into 5 groups that were defined using quintiles of KDI.

Cox proportional hazards models were used to analyze the relationship between age and sex-adjusted levels of these three kidney measures (i.e., 1/eGFR, ln (ACR × 100), and the KDI) and the first occurrence of the MACE outcome, death, and the kidney composite outcome. The shape of the relationship between these three kidney measures and the hazard of the three outcomes was assessed using Martingale residuals and spline plots. For these analyses the 3 kidney measures were expressed as Z-scores (calculated as an individual’s level minus the mean divided by the standard deviation). If a variable appeared to have a nonlinear relationship with an outcome, the corresponding model was re-estimated after including both the linear and squared variable in the model. The possibility of an interaction between 1/eGFR and ln (ACR × 100) with respect to these three outcomes was assessed by adding an interaction term to the models. Proportionality was assessed by plotting the log of negative log of the survival function against the log of time. Model performance was assessed using the C-statistic. Participants were censored at either the date of the final follow-up visit, the date of death, or the date of discontinuation from the trial.

All analyses were done using SAS software (version 9.4). This trial is registered with ClinicalTrials.gov, number NCT01394952.

## Results

The baseline clinical and biochemical characteristics of the 9,115 individuals, including 4222 (46.3%) women, who had a baseline ACR and a baseline eGFR are noted in Additional file 1: Table S1. Their mean (SD) age, diabetes duration and HbA1c levels were 66.2 (6.5) years, 10.5 (7.2) years and 7.3 (1.1%), respectively. The mean (SD) eGFR was 76.9 (22.8) ml/min/1.73 m^2^, and the mean (SD) inverse of eGFR (i.e., 1/eGFR) was 0.014 (0.007). The median ACR was 1.8 (IQR 0.7, 7.4), and the mean ln (ACR X 100) was 5.5 (SD 1.7) mg/mmol. The arithmetic mean (SD) of participants’ KDI values calculated as the geometric mean of their 1/eGFR and ln (100 X ACR) was 0.27 (0.08).

**Fig. 1:**
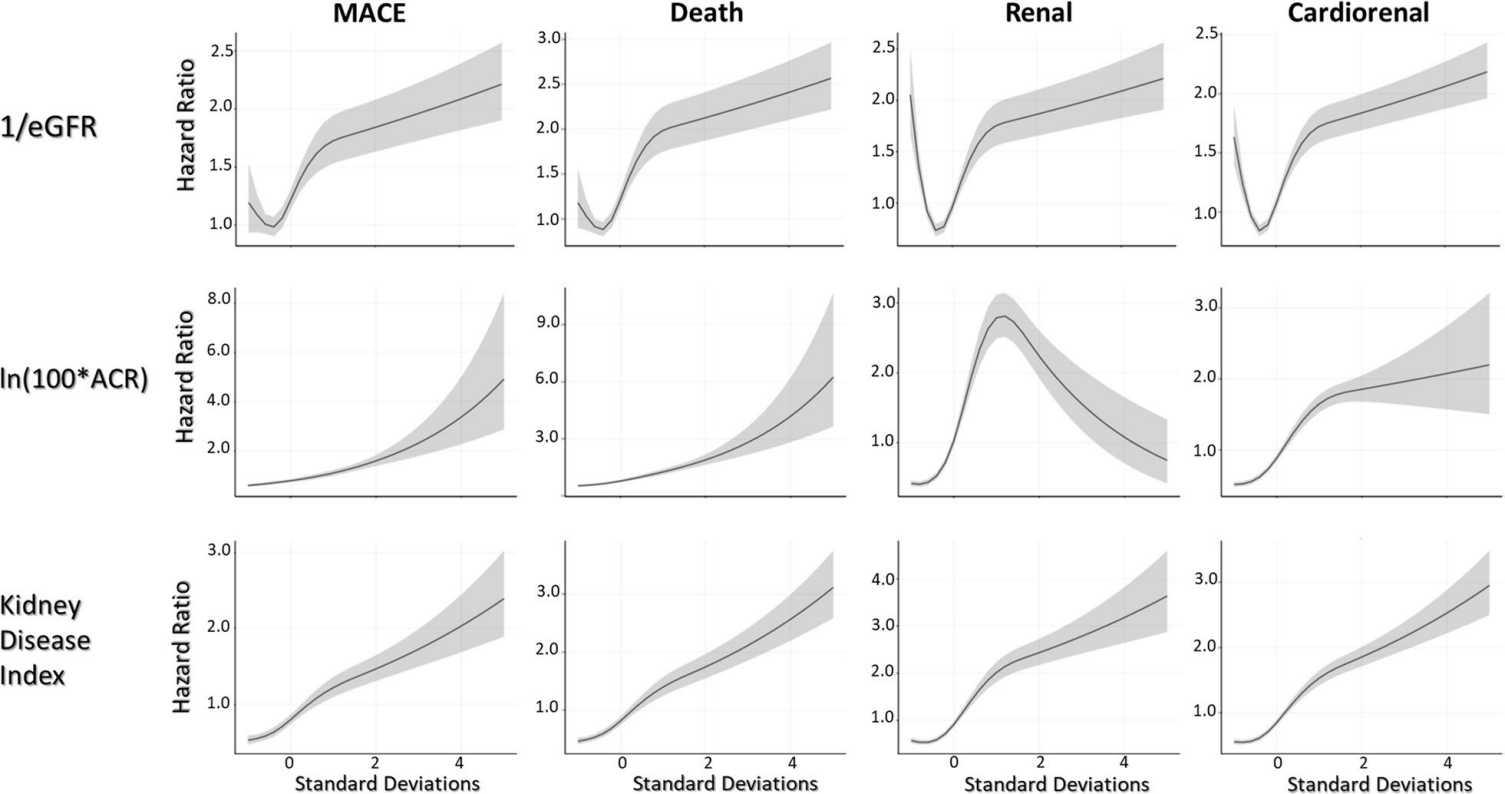
Spline curves illustrating the relationship between standard deviation-level increments in 1/eGFR, ln (100 × ACR), and the kidney disease index (i.e., the geometric mean of these 2 measures) that are displayed on the x axes, and hazards of the incident outcomes that are displayed on the y axis

The incidence of MACE, death, and the kidney composite outcome during follow-up was 2.51, 2.19 and 2.71 per 100 person-years respectively (Table 1). As noted in the Fig. 1 (spline) there was a non-linear relationship between 1/eGFR and MACE, death, and the kidney composite outcome. To account for this, hazard ratios for these 3 outcomes were estimated with a Cox model that included both a linear and a quadratic term for 1/eGFR. Using this model (Table 2), one standard deviation above the mean was associated with a MACE HR of 1.31 (95% CI 1.22, 1.40), a death HR of 1.52 (95% CI 1.42, 1.61), and a kidney composite HR of 1.34 (95% CI 1.25, 1.44). A significant interaction between 1/eGFR and sex suggested a higher hazard for women versus men (Additional file 1: Table S2) for MACE (interaction P = 0.012).

**Table 1:** Proportion of events and their incidence by fifths of the geometric mean of each person’s kidney disease index

	Overall N = 9115	Quintile 1 N = 1823	Quintile 2 N = 1823	Quintile 3 N = 1823	Quintile 4 N = 1823	Quintile 5 N = 1823	P
Median KDI (10—90)	0.26 (0.20–0.37)	0.20 (0.16–0.21)	0.23 (0.22–0.24)	0.26 (0.25–0.27)	0.30 (0.28–0.32)	0.37 (0.33–0.47)	
Median eGFR (10—90)	74.9 (48.8–106.8)	97.3 (73.0–126.2)	85.2 (67.2–109.9)	76.2 (59.6–98.2)	67.6 (51.7–86.9)	51.8 (33.5–69.9)	
Median ACR (10—90)	1.8 (0.3–29.8)	0.4 (0.1–1.3)	1.1 (0.4–3.7)	1.8 (0.6–9.0)	4.0 (1.0–27.0)	18.1 (2.2–109.4)	
MACE							
N (%)	1161 (12.7)	149 (8.2)	175 (9.6)	194 (10.6)	264 (14.5)	379 (20.8)	< 0.001
N/100py	2.51	1.54	1.84	2.08	2.90	4.43	
Death							
N (%)	1045 (11.5)	108 (5.9)	140 (7.7)	167 (9.2)	223 (12.2)	407 (22.3)	< 0.001
N/100py	2.19	1.09	1.43	1.74	2.36	4.56	
Kidney composite							
N (%)	1226 (13.5)	161 (8.8)	136 (7.5)	213 (11.7)	274 (15.0)	442 (24.2)	< 0.001
N/100py	2.71	1.68	1.43	2.31	3.08	5.55	

The kidney composite includes new macroalbuminuria, a sustained decline in eGFR ≥ 40%, or chronic kidney replacement therapy; the P value is from the chi-square test for trend

*GM* geometric mean; *10–90* the tenth and 90th percentile boundaries; *ACR* albumin-to-creatinine ratio; *eGFR* estimated glomerular filtration rate

**Table 2:** Age and sex-adjusted hazard with a one standard deviation higher level of markers of kidney disease

	Ln (100*ACR)	1/eGFR^a^	Interaction (P)^b^	KDI
Mean	5.47	0.014	N/A	0.27
Standard deviation	1.74	0.007	N/A	0.08
MACE (per 1 SD)	1.40 (1.32, 1.47)	1.31 (1.22, 1.40)	Negative (0.031)	1.27 (1.23, 1.31)
Death (per 1 SD)	1.53 (1.44, 1.62)	1.52 (1.42, 1.61)	Negative (0.018)	1.30 (1.27, 1.31)
Kidney composite (per 1 SD)	1.79 (1.68, 1.90)	1.34 (1.25, 1.44)	No Interaction	1.31 (1.28, 1.34)

Hazard rates are per 1 SD (standard deviation) higher level of the risk factor and are from the age and sex adjusted models for each variable

*ACR* urine albumin-to-creatinine ratio; *eGFR* estimated glomerular filtration rate; *KDI* kidney disease index

^a^As the relationship between 1/eGFR and the outcomes is not linear, the models include both 1/eGFR and the square of 1/eGFR as independent variables

^b^The interaction column notes whether there was an interaction between Ln(100*ACR) and 1/eGFR with respect to each of the 4 outcomes and is based on an age and sex adjusted Cox model that includes each term, an interaction term between the two, and a term that includes the square of 1/eGFR. The kidney composite includes new macroalbuminuria, a sustained decline in eGFR ≥ 40%, or chronic kidney replacement therapy

A non-linear relationship was also noted between Ln (100 × ACR) and the composite kidney outcome, whereas a linear relationship was noted between this variable and MACE, and death (Fig. 1). Thus, the hazard ratio for the composite kidney outcome was estimated with a Cox model that included both a linear and a quadratic term, whereas only a linear term was needed for the other outcomes. Using these models (Table 2) one standard deviation above the mean was associated with a MACE HR of 1.40 (95%CI 1.32, 1.47), a death HR of 1.53 (95%CI 1.44, 1.62), and a kidney outcome HR of 1.79 (95%CI 1.68, 1.90).

**Fig. 2:**
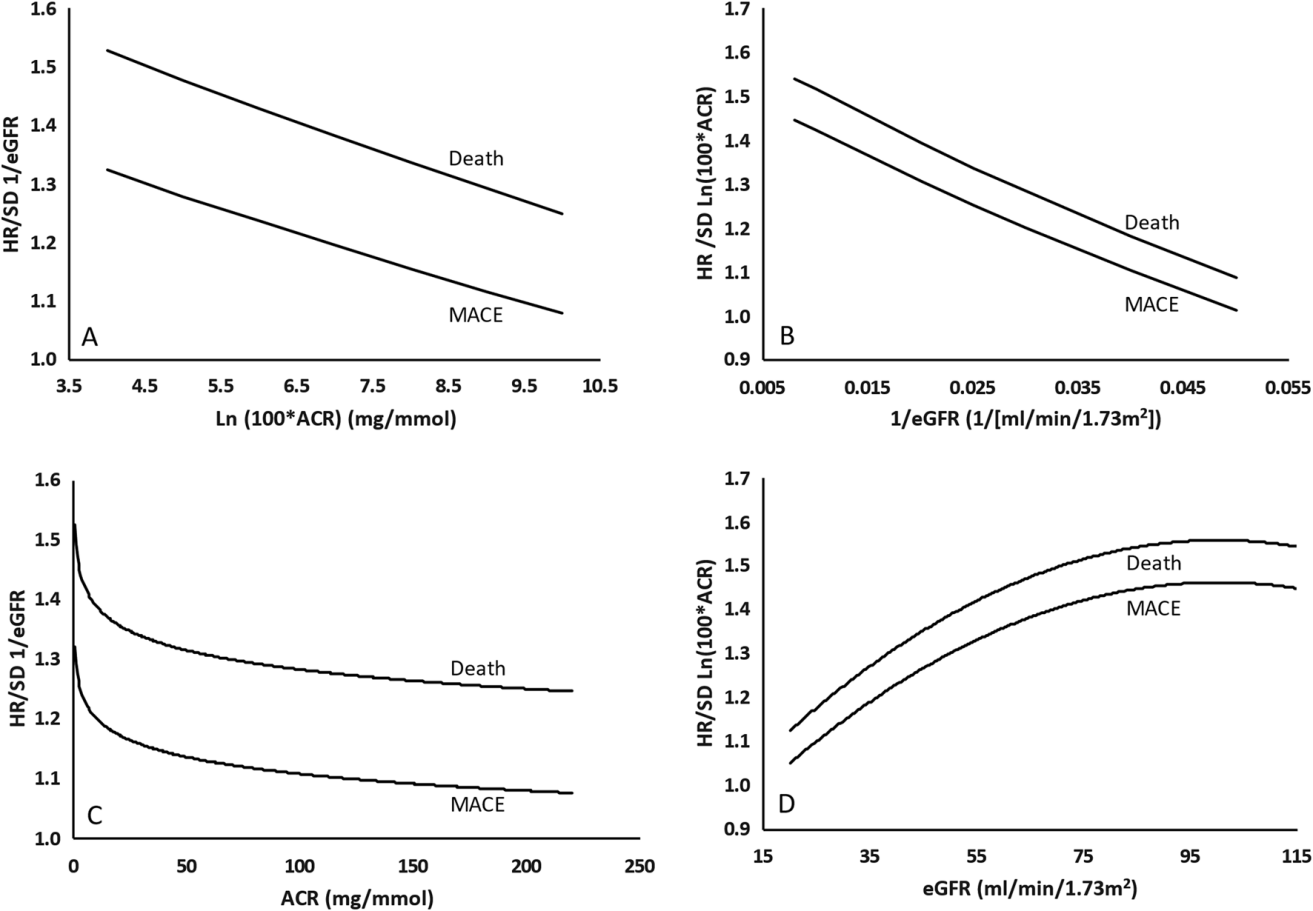
The figure illustrates the hazard of MACE, death, and the cardiorenal composite with a one standard deviation higher 1/eGFR at different levels of ln (100 × ACR) in **A** and at different levels of ACR in **C**. It also illustrates the hazard of these outcomes per standard deviation higher ln (100 × ACR) at different levels of 1/eGFR in **B**, and at different levels of eGFR in **D**. The figures were based on Cox models that included age, sex, 1/eGFR, ln(100 × ACR), the interaction of these two variables, and squared terms for 1/eGFR and ln (100 × ACR) where appropriate. *MACE* major adverse cardiovascular events; *eGFR* estimated glomerular filtration rate; *ACR* albumin-to-creatinine ratio

When both 1/eGFR and Ln (100 × ACR) were included in the same models, statistically significant, negative interactions for MACE (interaction P = 0.031) and death (interaction P = 0.018) were identified such that for these 2 outcomes (Table 2), the eGFR was a poor predictor in people with a high degree of albuminuria (Fig. 2a and c) and albuminuria was a poor predictor in people with a low eGFR (Fig. 2b and d). These interactions are illustrated in Fig. 2 which displays the predicted hazard ratio of these 2 outcomes at different levels of albuminuria and eGFR.

In contrast to both 1/eGFR and Ln (100 × ACR), a linear relationship was noted between the geometric mean of these 2 variables (the KDI) and all three outcomes (Fig. 1). Thus, every standard deviation higher KDI was associated with a 27% to 31% higher hazard of these outcomes (Table 2). A significant interaction between KDI and sex suggested a higher hazard for women versus men (Additional file 1: Table S2) for MACE (interaction P = 0.013).

As noted in Additional file 1: Table S1, those with higher KDI values were more likely to be women, and were older, had a longer duration of diabetes, and a higher systolic blood pressure, HbA1c and LDL cholesterol. They also had a higher prevalence of hypertension, cardiovascular disease, retinopathy and use of renin angiotensin system drugs, and a lower prevalence of tobacco and statin use. Higher fifths of KDI were also associated with a higher incidence of these outcomes (P for trend < 0.001) during follow-up (Table 1), such that participants in the highest fifth had an annual incidence of MACE, death, and the kidney composite outcome of 4.43, 4.56, and 5.55 per 100 person years respectively (Table 1).

The addition of the KDI to age and sex yielded areas under the receiver operating characteristics curves (i.e., C-statistics) that were greater than those for age and sex alone for MACE (0.63 versus 0.60), death (0.68 versus 0.63) and the renal composite (0.65 versus 0.52), and similar to those for the age and sex-adjusted eGFR and albuminuria components of the KDI when included in a multivariable model (Additional file 1: Table S3). The same pattern was noted when the KDI was assessed using data from the ORIGIN trial (Additional file 1: Table S4).

## Discussion

Diabetes guidelines recommend the annual assessment of the eGFR and the ACR to identify patients at high risk for serious outcomes [10]. In this epidemiologic analysis of 9,115 middle-aged men and women with type 2 diabetes and additional cardiovascular risk factors, both kidney function and albuminuria expressed as 1/eGFR and ln (100 × ACR) respectively predicted MACE, death, and a kidney composite outcome. Notably, there was a non-linear relationship between 1/eGFR and all these outcomes, and between albuminuria and the kidney composite outcome. Moreover, for MACE and death the prognostic value of each measure was diminished in the presence of higher levels of the other (Fig. 2). When both albuminuria and the eGFR were combined into a kidney disease index, calculated as a geometric mean of 1/eGFR and ln (100 × ACR), higher values were linearly related to all 3 outcomes. Moreover, the age and sex- adjusted prognostic value of the KDI was similar to the full complex model that accounted for both variables, their non-linear relationships, and their interactions with each other.

Many epidemiologic analyses evaluated multivariable prediction models for diabetes-related kidney outcomes and reported excellent performance for models that include many variables in addition to eGFR and/or ACR [11]. Some have identified both the eGFR and ACR as important indices for kidney outcomes [12–15], cardiovascular outcomes [16–18], and death [14–16]. However, few have formally interrogated the shape of the relationship between these 2 variables and kidney outcomes as well as cardiovascular outcomes and death. The observation of a non-linear relationship between the eGFR variable and all four outcomes in the REWIND data is consistent with the findings of a large meta-analysis published in 2012 [15].

Both the eGFR and albuminuria are generally viewed as independent determinants of these outcomes. Indeed, the 2012 Kidney Disease Improving Global Outcomes (KDIGO) guidelines graphic which displays albuminuria and eGFR categories on 2 axes [2] (Additional file 1: Figure S1) implies an orthogonal or independent relationship between these 2 variables and suggests that highly abnormal levels of both confer a worse prognosis for various outcomes than abnormal levels of only one alone. These analyses show that this is indeed the case for the kidney outcome. However, the observed negative interactions illustrated in Fig. 2 in which high degrees of either of these risk factors (albuminuria or kidney dysfunction) were poor predictors of MACE and death in the presence of high degrees of the other, suggests that they are not independent risk factors for these other outcomes. Most epidemiologic analyses have not tested for such interactions. When they were assessed, a similar negative interaction for cardiovascular events was reported in one publication [17], but not another [16].

The non-linear relationships and interactions noted above plus the fact that both the eGFR and albuminuria are prognostically relevant for future serious outcomes highlight the potential value of a metric that combines the information contained in both the eGFR and the ACR. The KDI is such a metric. Its linear relationship to the 3 outcomes illustrated in Fig. 1, its straightforward calculation, and the fact that its area under the receiver operating characteristics curve is similar to that of complicated models that include interactions and squared terms, highlight its possible utility as a simple prognostic marker for kidney outcomes as well as cardiovascular outcomes and death.

Strengths of these exploratory, post hoc analyses include the international nature of these data, the fact that the participants are similar to people with diabetes in the general population [19], the long-term follow-up, the high number of outcomes, the fact that all clinical outcomes were adjudicated, and consistent findings when the new index was applied to a different cohort from the ORIGIN trial. The absence of a central laboratory for the urine and serum measurements is a limitation common to many epidemiologic studies of kidney function [20] that is mainly mitigated by the large sample size. These analyses are also limited by the fact that they were not prespecified, and the fact that they were conducted in middle-aged and older people with diabetes and additional cardiovascular risk factors who consented to participate in a long-term randomized controlled trial. They therefore may not be applicable other populations.

## Conclusion

The eGFR and ACR are routinely measured risk factors for kidney and cardiovascular outcomes. The observation that these risk factors were not linearly related to these outcomes and were not independent of each other for predicting MACE and death highlights their complex relationship to each other and to these outcomes. The KDI is a composite measure of these two risk factors. It combines information from both risk factors and has a simple linear relationship to all three outcomes. Moreover, its ability to predict these three outcomes was similar to the ability of complex models that included the eGFR and ACR, nonlinear terms and interaction terms to predict these outcomes. The KDI could therefore simplify the identification of the highest risk individuals who are most likely to benefit from preventive therapies. Its utility as a risk stratification tool should be assessed and confirmed in future epidemiologic studies and clinical trials.

## Supplementary Information

**Additional file 1: Table S1.** Distribution of Baseline Characteristics Across Fifths of the Kidney Disease Index*. **Table S2.** Age-adjusted Hazard of Different Outcomes According to Sex. **Table S3.** C-statistics for the Age and Sex-Adjusted Models. **Table S4.** C-statistics for the Age and Sex-Adjusted Models Using ORIGIN Data. **Figure S1.** The prognosis of CKD by GFR and Albuminuria Categories (KDIGO 2012) is indicated by the risk categories in the cells of the figure.

## Data Availability

The REWIND data sharing policy is described in the Supplement.

## Abbreviations

REWIND: Researching Cardiovascular Events with a Weekly Incretin in Diabetes
eGFR: Estimated glomerular filtration rate
ACR: Albumin-to-creatine ratio
KDI: Kidney disease index
MACE: Major adverse cardiovascular events
