# Supplementary material for: A novel kidney disease index reflecting both the albumin-to-creatinine ratio and estimated glomerular filtration rate, predicted cardiovascular and kidney outcomes in type 2 diabetes
Source: Cardiovasc Diabetol. 2022 Aug 22;21:158. doi: 10.1186/s12933-022-01594-6 (PMC9396793; doi:10.1186/s12933-022-01594-6)
Supplement: Supplementary file 1 — Additional file 1: Table S1. Distribution of Baseline Characteristics Across Fifths of the Kidney Disease Index*. Table S2. Age-adjusted Hazard of Different Outcomes According to Sex. Table S3. C-statistics for the Age and Sex-Adjusted Models. Table S4. C-statistics for the Age and Sex-Adjusted Models Using ORIGIN Data. Figure S1. The prognosis of CKD by GFR and Albuminuria Categories (KDIGO 2012) is indicated by the risk categories in the cells of the figure. [file 12933_2022_1594_MOESM1_ESM.docx]

**Supplement**

**A Novel Kidney Disease Index Reflecting both the Albumin-to-Creatinine Ratio and Estimated Glomerular Filtration Rate, Predicted Cardiovascular and Renal Outcomes in Type 2 Diabetes**

**Contents**

Table S1: Distribution of Baseline Characteristics Across Fifths of the Kidney Disease Index 2

Table S2: Age-adjusted Hazard of Different Outcomes According to Sex 3

Table S3: C-statistics for the Age and Sex-Adjusted Models 4

Table S4: C-statistics for the Age and Sex-Adjusted Models Using ORIGIN Data

Figure S1: Prognosis of CKD by GFR and Albuminuria Categories (KDIGO 2012) 6

Data Sharing Policy 7

| **Table S1: Distribution of Baseline Characteristics Across Fifths of the Kidney Disease Index*** | | | | | | | |
| --- | --- | --- | --- | --- | --- | --- | --- |
|  | **OVERALL**  **N = 9115** | **Quintile 1**  **N = 1823** | **Quintile 2**  **N = 1823** | **Quintile 3**  **N = 1823** | **Quintile 4**  **N = 1823** | **Quintile 5**  **N = 1823** | **P-value** |
| Geometric Mean | 0.27 (0.08) | 0.19 (0.03) | 0.23 (0.01) | 0.26 (0.01) | 0.30 (0.01) | 0.39 (0.08) |  |
| eGFR (ml/min/1.73m^2^) | 76.9 (22.8) | 98.3 (20.5) | 87.3 (17.3) | 78.1 (15.5) | 68.6 (13.7) | 51.9 (13.9) | <0.0001 |
| Median ACR | 1.8 (0.7-7.4) | 0.4 (0.2-0.8) | 1.1 (0.6-2.0) | 1.8 (1.0-4.0) | 4.0 (1.8-9.9) | 18.1 (5.4-49.1) | <0.0001 |
| 1/eGFR (ml/min/1.73m^2^) | 0.014 (0.007) | 0.011 (0.002) | 0.012 (0.002) | 0.013 (0.003) | 0.015 (0.003) | 0.021 (0.013) | <0.0001 |
| Ln(100*ACR) (mg/mmol) | 5.5 (1.7) | 3.7 (1.1) | 4.7 (0.9) | 5.4 (1.1) | 6.2 (1.3) | 7.4 (1.5) | <0.0001 |
| Age (years) | 66.2 (6.5) | 64.5 (5.9) | 65.4 (6.2) | 66.0 (6.1) | 66.9 (6.6) | 68.4 (7.0) | <0.0001 |
| Female | 4222 (46.3) | 812 (44.5) | 826 (45.3) | 821 (45.0) | 866 (47.5) | 897 (49.2) | 0.022 |
| Diabetes duration (years) | 10.5 (7.2) | 9.5 (6.6) | 9.6 (6.6) | 10.3 (6.8) | 10.7 (7.4) | 12.5 (8.2) | <0.0001 |
| Current Tobacco Use | 1288 (14.1) | 327 (17.9) | 258 (14.2) | 254 (13.9) | 256 (14.0) | 193 (10.6) | <0.001 |
| Hypertension | 8500 (93.3) | 1653 (90.7) | 1675 (91.9) | 1702 (93.4) | 1716 (94.1) | 1754 (96.2) | <0.001 |
| Prior Cardiovascular Disease | 2842 (31.2) | 553 (30.3) | 550 (30.2) | 542 (29.7) | 597 (32.7) | 600 (32.9) | 0.095 |
| Prior Cardiovascular Event | 1854 (20.3) | 350 (19.2) | 365 (20.0) | 342 (18.8) | 406 (22.3) | 391 (21.4) | 0.041 |
| Diabetic Retinopathy | 824 (9.0) | 123 (6.7) | 139 (7.6) | 158 (8.7) | 170 (9.3) | 234 (12.8) | <0.001 |
| Statin use | 6007 (65.9) | 1260 (69.1) | 1237 (67.9) | 1205 (66.1) | 1175 (64.5) | 1130 (62.0) | <0.001 |
| ACE inhibitor/ARB use | 7416 (81.4) | 1431 (78.5) | 1440 (79.0) | 1486 (81.5) | 1507 (82.7) | 1552 (85.1) | <0.001 |
| Body Mass Index(kg/m^2^) | 32.3 (5.7) | 32.2 (5.5) | 32.4 (5.7) | 32.4 (5.6) | 32.3 (6.0) | 32.3 (5.8) | 0.6412 |
| Systolic BP (mm Hg) | 137.3 (16.8) | 134.6 (15.9) | 135.8 (16.4) | 136.7 (16.0) | 138.1 (17.0) | 141.2 (18.1) | <0.0001 |
| Diastolic BP (mm Hg) | 78.5 (9.8) | 78.3 (9.5) | 78.3 (9.6) | 78.6 (9.7) | 78.9 (9.7) | 78.4 (10.6) | 0.3296 |
| Heart Rate (beats/min) | 71.4 (10.9) | 71.1 (10.3) | 70.9 (10.6) | 71.1 (10.7) | 72.1 (11.2) | 71.9 (11.5) | 0.0010 |
| HbA1c (%) | 7.3 (1.1) | 7.2 (1.0) | 7.3 (1.0) | 7.3 (1.0) | 7.4 (1.1) | 7.5 (1.1) | <0.0001 |
| LDL cholesterol (mmol/L) | 2.6 (1.0) | 2.5 (1.0) | 2.5 (1.0) | 2.5 (1.0) | 2.6 (1.0) | 2.7 (1.0) | <0.0001 |
| Means (standard deviations), medians (interquartile ranges) and numbers (%) are shown. Cardiovascular disease – myocardial infarction, ischemic stroke,  unstable angina with electrocardiogram changes, myocardial ischemia on imaging or stress test, or coronary, carotid, or peripheral revascularization; Cardiovascular event – myocardial infarction or ischemic stroke; eGFR – estimated glomerular filtration rate; ACR – albumin-to-creatinine ratio; ACE – angiotensin converting enzyme; ARB – angiotensin receptor blocker; BP – blood pressure; IQR – inter-quartile range. The P value is from the chi-square test for trend. *The kidney disease index for each participant is the geometric mean of 1/eGFR and Ln (100*ACR); the arithmetic mean (SD) of its level in the REWIND population was 0.27 (0.08). | | | | | | | |

| **Table S2: Age-adjusted Hazard of Different Outcomes According to Sex** | | | | | | |
| --- | --- | --- | --- | --- | --- | --- |
|  | **1/eGFR per SD** | **P Int** | **Ln (100*ACR) per SD** | **P Int** | **KDI per SD** | **P Int** |
| **MACE** |  |  | . |  |  |  |
| All | 1.31 (1.22, 1.40) |  | 1.40 (1.32, 1.47) |  | 1.27 (1.23, 1.31) |  |
| Women | 1.36 (1.26, 1.46) | 0.012 | 1.34 (1.22, 1.47) | 0.30 | 1.37 (1.29, 1.45) | 0.013 |
| Men | 1.26 (1.17, 1.36) |  | 1.43 (1.33, 1.53) |  | 1.25 (1.20, 1.30) |  |
| **Death** | . |  |  |  |  |  |
| All | 1.52 (1.42, 1.61) |  | 1.53 (1.44, 1.62) |  | 1.30 (1.27, 1.31) |  |
| Women | 1.54 (1.44, 1.65) | 0.29 | 1.58 (1.44, 1.74) | 0.37 | 1.32 (1.27, 1.37) | 0.34 |
| Men | 1.49 (1.39, 1.60) |  | 1.50 (1.40, 1.61) |  | 1.29 (1.24, 1.33) |  |
| **Renal Composite** |  |  |  |  |  |  |
| All | 1.34 (1.25, 1.44) |  | 1.79 (1.68, 1.90) |  | 1.31 (1.28, 1.34) |  |
| Women | 1.33 (1.24, 1.44) | 0.63 | 1.70 (1.55, 1.86) | 0.15 | 1.31 (1.26, 1.37) | 0.93 |
| Men | 1.36 (1.25, 1.47) |  | 1.86 (1.72, 2.01) |  | 1.31 (1.27, 1.35) |  |
| KDI – kidney disease index estimated as the geometric mean of Ln(100*ACR) & 1/eGFR; the renal composite includes new macroalbuminuria, a sustained decline in eGFR ≥ 40%, or chronic renal replacement therapy; the cardiorenal composite includes MACE, death, and the renal composite | | | | | | |

| **Table S3: C-statistics for the Age and Sex-Adjusted Models** | | | | | |
| --- | --- | --- | --- | --- | --- |
|  | **Age and Sex Alone** | **Age, Sex, ln(100*ACR)** | **Age, Sex, 1/eGFR^1^** | **Age, Sex, ln(100*ACR), 1/eGFR, Interactions^2^** | **Age, Sex, Kidney Disease Index (KDI)** |
| MACE | 0.60 (0.58, 0.61) | 0.64 (0.62, 0.65) | 0.62 (0.60 ,0.63) | 0.64 (0.63, 0.66) | 0.63 (0.62, 0.65) |
| Death | 0.63 (0.61, 0.65) | 0.68 (0.66, 0.70) | 0.66 (0.64, 0.68) | 0.69 (0.67, 0.71) | 0.68 (0.66, 0.69) |
| Renal Composite | 0.52 (0.50, 0.54) | 0.69 (0.67, 0.70)^3^ | 0.55 (0.54, 0.57) | 0.69 (0.68, 0.71) | 0.65 (0.63, 0.67) |
| C statistics with 95% confidence intervals are shown. The renal composite includes new macroalbuminuria, a sustained decline in eGFR ≥ 40%, or chronic renal replacement therapy. ^1^ All 3 models included the linear and squared term. ^2^ The models included both linear and squared terms for 1/eGFR (and the linear and squared terms for ln[100 x ACR] for the renal composite) as well as the interaction terms of these 2 variables for those outcomes with an interaction (i.e., MACE and death); ^3^ This model included the linear and squared term. Kidney disease index – geometric mean of 1/eGFR and Ln (100 x ACR) | | | | | |

| **Table S4: C-statistics for the Age and Sex-Adjusted Models Using ORIGIN Data** | | | |
| --- | --- | --- | --- |
|  | **Age and Sex Alone** | **Age, Sex, ln(1000*ACR), ln (1000/eGFR), Interactions^1^** | **Age, Sex, Kidney Disease Index (KDI)^2^** |
| MACE | 0.61 (0.59, 0.62) | 0.64 (0.62, 0.65) | 0.63 (0.62, 0.65) |
| Death | 0.64 (0.63, 0.66) | 0.68 (0.67, 0.69) | 0.68 (0.66, 0.69) |
| Renal Composite | 0.54 (0.52, 0.55) | 0.64 (0.62, 0.65) | 0.64 (0.62, 0.65) |
| C statistics with 95% confidence intervals are shown. The renal composite includes doubling of serum creatinine, worsening of albuminuria category, chronic kidney replacement therapy or death due to kidney failure. ^1^ the interaction of ln(1000*ACR) and ln (1000/eGFR) is included in the model. ^2^Kidney disease index = geometric mean of ln(1000*ACR) and ln (1000/eGFR) | | | |

**Figure S1:** The prognosis of CKD by GFR and Albuminuria Categories (KDIGO 2012) is indicated by the risk categories in the cells of the figure.

|  |  |  | **Albuminuria [mg/g (mg/mmol)]** | | |
| --- | --- | --- | --- | --- | --- |
|  |  |  | **A1 (Normo-)** | **A2 (Micro-)** | **A3 (Macro-)** |
|  |  |  | **<30 (< 3)** | **30-300 (3-30)** | **>300 (>30)** |
| **eGFR Stage [ml/min/1.73m^2^]** | **G1** | **≥ 90** | *Low* | *Moderate* | *High* |
|  | **G2** | **60-89** | *Low* | *Moderate* | *High* |
|  | **G3a** | **45-59** | *Moderate* | *High* | *Very High* |
|  | **G3b** | **30-44** | *High* | *Very High* | *Very High* |
|  | **G4** | **15-29** | *Very High* | *Very High* | *Very High* |
|  | **G5** | **<15** | *Very High* | *Very High* | *Very High* |

**Population Health Research Institute (PHRI) Data Sharing Policy**

Effective Date: July 27, 2018

Data will be disclosed only upon request and approval of the proposed use of the data by a review committee. Membership in the review committee will be determined by the executive leadership of the study. Generally only those requests made by a journal's statistician regarding the data related to the results of the publication will be considered unless the review committee sees high merit in other requests. The following principles will apply to requests:

1. The review committee will have established criteria to review the request to ensure that patient privacy and rights, and PHRI data and research integrity can be maintained with the sharing of the data. This includes (but is not limited to) demonstrated competence related to data security and data analysis by the investigator requesting access. The review committee will also ensure that provision of data to external parties does not contravene any prior agreement with any other parties.
2. PHRI will make individual participant data available, including data dictionaries, within the requirements and/or restrictions of REB/IRB and subject to the conditions set forth in the consent forms of the study. Data provided will be limited to data which underlies the results in the main publication after de-identification. Any analyses and publications should be reviewed and approved by PHRI before publication to ensure that the analyses are accurate and that the publication is not misleading.
3. The study protocol and the statistical analysis plan for analysis of the primary results will be shared.
4. For those requests that originate from concerns expressed by the journal about the data or statistical analyses, the data will be available to the journal statisticians in a timely manner.
5. Data can be disclosed for all other requests from 2 years after the main paper is published plus 6 additional months for each year of study conduct. However, there will be a maximum of 7 years to the time limit restriction.
6. Data will be shared to achieve the objective in the approved proposal with no additional analysis permitted without approval. Only proposals for analyses that do not compete with ongoing analyses or analyses proposed by study investigators will be approved.
7. Data will be made available by one of the following mechanisms. 1) The Statistics Department at PHRI can perform the analysis in accordance with the SAP provided by the investigator and under his/her supervision or 2) Arrangement can be made to transfer the data to a secure location using a process that has been verified by the Director of Statistics at PHRI.
8. Every proposal must identify and provide funding sufficient to defray the cost of data preparation, storage, transfer and analysis for the organization incurring these costs (this may include studies not fully funded from external sources i.e. industry or peer review grants). On occasions where the new analyses proposed are of sufficient scientific interest to PHRI, then a collaborative agreement for joint analyses and publication can be developed and charges may be reduced.
9. The data will be provided for a specified time limit that can allow completion of the analyses that is proposed. At the end of the proposed analyses, the requesting party undertakes to return or destroy the data base provided and provide written documentation of this.

**References**

1. Taichman DB, Sahni, P, Pinborg A et al.Data Sharing Statements for Clinical Trials - A Requirement of the International Committee of Medical Journal Editors. N Engl J Med 2017; 376: 23:2277-9.
2. International Consortium of Investigators for Fairness in Trial Data. Devereaux PJ, Guyatt G, Gerstein H, Connolly S, Yusuf S. Toward Fairness in Data Sharing. N Engl J Med 2016;375:405-7.

Supplemental Endorsers of Article listed in 2
